# The Use of Informative Priors in Bayesian Modeling Age-at-death; a Quick Look at Chronological and Biological Age Changes in the Sacroiliac Joint in American Males

**DOI:** 10.3934/publichealth.2017.3.278

**Published:** 2017-06-07

**Authors:** Kanya Godde

**Affiliations:** 1 Sociology/Anthropology Department, University of La Verne, La Verne, CA 91750, USA; 2 Department of Anthropology, University of Tennessee, Knoxville, Knoxville, TN, USA

**Keywords:** Lovejoy method, auricular surface, transition analysis, pelvis, survivorship

## Abstract

The aim of this study is to examine how well different informative priors model age-at-death in Bayesian statistics, which will shed light on how the skeleton ages, particularly at the sacroiliac joint. Data from four samples were compared for their performance as informative priors for auricular surface age-at-death estimation: (1) American population from US Census data; (2) county data from the US Census data; (3) a local cemetery; and (4) a skeletal collection. The skeletal collection and cemetery are located within the county that was sampled. A Gompertz model was applied to compare survivorship across the four samples. Transition analysis parameters, coupled with the generated Gompertz parameters, were input into Bayes' theorem to generate highest posterior density ranges from posterior density functions. Transition analysis describes the age at which an individual transitions from one age phase to another. The result is age ranges that should describe the chronological age of 90% of the individuals who fall in a particular phase. Cumulative binomial tests indicate the method performed lower than 90% at capturing chronological age as assigned to a biological phase, despite wide age ranges at older ages. The samples performed similarly overall, despite small differences in survivorship. Collectively, these results show that as we age, the senescence pattern becomes more variable. More local samples performed better at describing the aging process than more general samples, which implies practitioners need to consider sample selection when using the literature to diagnose and work with patients with sacroiliac joint pain.

## 1. Introduction

It has been established that skeletal age indicators alone do not adequately describe and capture chronological age [1]–[4], although they may be decent indicators of biological age. In order to contend with the differences in age types, the application of Bayesian statistics, which takes into account a prior age-at-death distribution, can help to model biological age so that it captures chronological age well (e.g., 1–7). One of the first considerations for trying to ascertain chronological age from the skeleton with statistical analysis is identifying an appropriate prior sample from which to draw an age distribution in Bayesian analysis.

Populations age differently skeletally [1],[3],[7]–[9], which necessitates finding a prior sample with a relatively similar age distribution, usually from a source population comparable to the target population (the population to which a practitioner seeks to assign chronological ages). The informative prior can also be derived from the target population, wherein a portion of the population from which chronological age is to be calculated can be used as the informative prior, and is referred to as the forensic approach in the literature (c.f., 2). The knowledge of this prior age distribution in a Bayesian analysis strengthens the accuracy of age estimations assigned to different phases of a skeletal indicator, although past work has established that there is some leeway in the fit of the prior sample to the target sample from which age is to be estimated [1].

Biological indicators of age are important to understand for historic and forensic populations. Knowledge about how these indicators change as we age can inform practitioners of joint changes that affect older adults, which in turn apprises application of age-at-death estimation methods. In particular, the sacroiliac joint is a source of pain for adults as they age due to the development of ankylosing spondylitis and changes to the cartilage that cushions the joint. As one ages, the sacroiliac joint transitions to become rougher and more irregular [10],[11]. Changes to the cartilage is asymmetric; degeneration of the iliac surface occurs at an earlier age than the sacral surface [11]. Movement at the joint can become restricted when a person is in their 50s due to cartilaginous changes that result in an increase of collagen and fibrous ankylosing [11]. These senescent alterations continue into a person's 70s [11]. The morphological changes at the sacroiliac joint are the features that are captured in biological indicators of age; each phase describes a set of characteristics that we transition through as we age. Two popular methods of sacroiliac, or auricular surface, skeletal aging include an 8-phase [10] system and a composite score method [12]. In this research, I utilize the Lovejoy et al. [10] 8-phase method as a measure of the applicability of prior samples.

It is of interest in this research to look at different representative levels of prior samples to ascertain which is the most appropriate for selection in Bayesian analyses of age-at-death. The potential pools of age-at-death structure data can be found in skeletal collections, cemeteries, county records, and country records, to name a few. Here, I examine the age-at-death demographics from an American skeletal collection developed mostly from willing, local donors, a cemetery local to the skeletal collection, the county in which the collection and cemetery reside, and the country in which the collection, cemetery, and, county are located. The auricular surface indicators were recorded from individuals in the skeletal collection. Thus, the forensic approach, wherein a subset of the target sample is used as an informative prior, will be called upon in this analysis.

This paper takes a biodemographic approach to investigating age and age-related changes in the skeleton. While this paper focuses on skeletal indicators of senescence, these data inform us of the aging processes humans undergo, which provides insight into longevity. The age-at-death distribution of several samples are examined and tested for differences that might impact age-at-death information derived from the skeleton, which impacts our understanding of biological and chronological age. Based on the literature, I hypothesize there will be small differences in the age-at-death distributions of the four samples. The performance of each sample will be measured by the calculated accuracy of auricular surface to capture chronological age from biological age, which supports the aim of this article.

## 2. Materials and Methods

The age-at-death distribution of four American male samples was modeled. The US Census dataset provided information for two of the samples: (1) the age-at-death of all adult (age 18 and older) American males who died between 1999 and 2013 (N = 2,197,657,812); and (2) the age-at-death of adult Knox County, Tennessee American males who died between 1999 and 2013 (N = 2,269,950). All causes of death regardless of whether an autopsy was performed, were considered. No restrictions were placed upon the data obtained from the US Census other than gender and Knox County residency.

The third sample (N = 2,275) is an age-at-death distribution from a local cemetery that represents Knox County residents. The cemetery was not restricted to a particular type of resident (e.g., a veterans' cemetery), which should provide a relatively unbiased sample of Knox county residents as each resident has an equal probability of being buried in the cemetery. These data were obtained from “Find a Grave”, an online research dataset, which allows for use of cemetery data for “scholarly historical research.” These results are applicable to historical and contemporary Americans. The date of death, date of birth, and name of the interred individuals were obtained from headstones and are accessible online. The data were screened for duplicate entries and names assessed for gender. Names were assigned probable genders based on the knowledge of gender-name association for the region. Individuals with gender neutral names were eliminated. Only males age 18 and over were included.

The final sample (N = 1,022) is comprised of individuals from the William M. Bass Donated Skeletal Collection (Donated Collection), housed at the University of Tennessee, Knoxville. The Anthropological Research Facility that is associated with the Donated Collection was started in 1980 by Dr. William Bass and represents local, regional, and global donations. Local donors mostly comprise the collection [13]. There is documentation of date of birth, date of death, and gender for the individuals accessioned in the collection. The documentation can be self-reported, family-reported, or reported by medical examiners [13]. Only ages from males 18 and older were utilized.

Senescence changes of the auricular surface were captured through visually inspecting the morphology and classifying the stage of the indicator into one of the 8 phases in the Lovejoy et al. method [10]. These phases are sequential and capture the biological aging process of the sacroiliac joint. The underlying theory of the method is that as the phases increase, so does the age [10]. The data were collected from adult males with documentation of age and sex and were provided by Dr. Samantha M. Hens. Conflicting indicators, e.g., a mixture of youthful and older traits, were dealt with by assigning a higher phase if older characteristics were present, despite the simultaneous preservation of youthful features [2]. Age-at-death for the observed individuals was not estimated using the age range associated with each phase in the Lovejoy et al. method [10]. Instead, the documented age-at-death was used in all statistical modeling to more closely align the phase and age-at-death.

A Gompertz survivorship model was ran on the ages of the individuals in all four samples, which estimates the mortality and senescence parameters:

h(t)=α(expβt) (1)

where *h(t)* is the death rate adjusted by 18 years (the minimum age of this sample), *α* is the mortality parameter and *β* the senescence parameter. These parameters represent the values used for the informative prior in Bayesian analysis. A single plot was generated with the survivorship curve for each of the four samples to compare the survivorship distribution.

In order to compare the performance of each sample to estimate chronological age from biological age indicators, the accuracy of each method must be ascertained. To do this, the transition analysis parameters that provide the age at which an individual transitions from phase to phase in the auricular surface method for this sample were obtained from Hens and Godde [2]. The transition analysis parameters are generated from a cumulative probit model utilizing the natural log of age and the auricular surface phase scored on each individual (for more information on this procedure see Hens and Godde [2]).

To calculate age ranges associated with each auricular surface phase, the transition analysis parameters (age at which an individual transitions from phase to phase) are combined with the aforementioned Gompertz parameters (mortality and senescence), which represent the informative prior's age-at-death distribution, in Bayes Theorem. Probability density functions (PDF) are integrated into Bayes theorem:

$$\mathrm{Pr}\left(a|c_{j}\right)=\frac{\mathrm{Pr}\left(c_{j}|a\right)f\left(a\right)}{\int_{0}^{\infty }\mathrm{Pr}\left(c_{j}|x\right)f\left(x\right)dx}$$ (2)

where *Pr*(*c_j_|a*) is the probability of an individual who dies at age *a* would be in the *j^th^* stage of skeletal morphology (phase) *c*. PDF are used to generate highest posterior densities and highest posterior density ranges (HPDR), which correspond to coverage, or a provided age range. In other words, 90% coverage is utilized here, which means the age ranges provide 90% coverage of the individuals who scored a particular phase. Thus, for phase 2, 90% of the individuals who scored a phase 2 should have an age-at-death that falls in the HPDR for phase 2. Cumulative binomial tests examined the knowledge of whether the coverage provided from the HPDR will meet the specified coverage. A small hold out sample of individuals from the Donated Collection, that were not utilized to construct the HPDRs, were assessed with cumulative binomial tests using age-at-death and assigned phase. This ascertained whether the 90% HPDR coverage held true in a separate sample.

The Bayesian approach of this analysis stems from the known prior age-at-death information from each of the four samples. The protocol for generating the age ranges follows the work of Konigsberg and colleagues [3] that was later employed by Godde and Hens [1],[2],[4]. All analyses were completed in R [14].

## 3. Results

**Figure 1.**
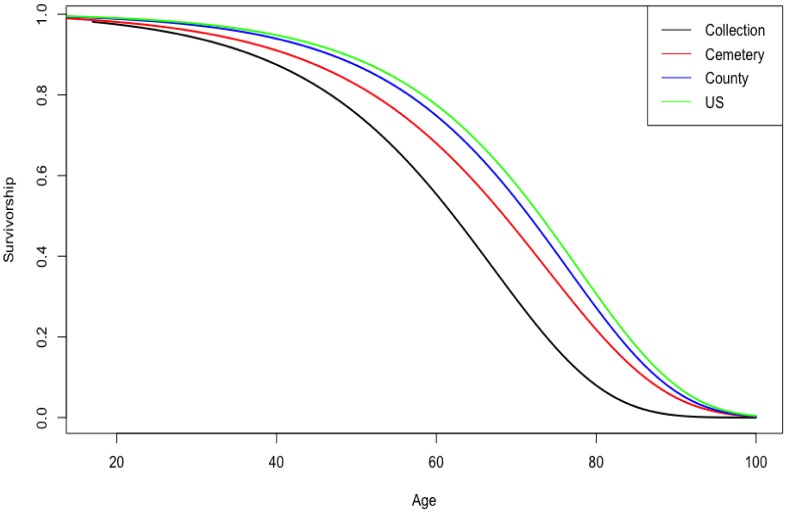
Gompertz survivorship of four male American samples.

The mortality and senescence parameters are listed in Table 1. Figure 1 depicts the different survivorship curves for the four samples. The four curves are relatively similar for the county and country data. However, the cemetery and skeletal collections demonstrate a younger survivorship than the county or country samples. Moreover, there are shape differences in older ages (e.g., age 60 and age 80) of the cemetery and skeletal collections in comparison to the county and country estimates, with the skeletal collection having the most dissimilar survivorship of the four samples. The US and county census curves run very close with little differentiation, particularly in the younger ages (18–30 years old).

**Table 1.** List of Gompertz mortality and senescence parameters.

*Sample*	*α*	*β*
Donated Collection	0.000570474	0.072138553
Cemetery	0.000451549	0.06799743
Knox County	0.000248608	0.074624255
US	0.000201514	0.076328717

The HPDRs (Table 2) showed the greatest precision of age ranges using the Donated Collection ages-at-death as the prior; thus the forensic method performed the best of the four prior samples. The younger ages boasted more narrow age ranges across the samples, with the Donated Collection providing the narrowest ranges in the higher phases, which correspond to higher ages. Despite the broad ranges inherent in the higher phases, they were also the least accurate age ranges (Table 3) with a higher failure rate in phases V-VIII vs. the lower phases. Cumulative binomial tests demonstrated that the HPDR performed lower than expected for 90% coverage (Table 4).

**Table 2.** Computed 90% HPDR for each sample using the Lovejoy et al. [10] method.

*Phase*	*Donated Collection*	*Cemetery*	*Knox County*	*US*
I	18.00–32.29	18.00–32.05	18.00–32.50	18.00–32.63
II	18.30–48.68	18.15–48.10	18.33–49.65	18.38–50.09
III	24.42–62.69	24.07–62.53	24.49–64.70	24.58–65.37
IV	31.16–76.08	30.54–77.28	31.50–80.35	31.76–81.42
V	41.74–87.81	41.39–91.27	43.29–94.24	43.86–95.46
VI	52.23–95.28	52.98–100.49	55.42–102.73	56.25–103.86
VII	61.02–99.89	63.03–106.09	65.49–107.63	66.44–108.63
VIII	70.19–104.73	74.09–112.12	76.38–112.90	77.43–113.78

**Table 3.** Distribution of individuals within each age range for cumulative binomial tests.

*Sample*	*Phase*	*Number of Successes*	*Number of Failures*	*Probability of Success*
Donated Collection	I	4	0	1.00
	II	19	1	0.95
	III	18	0	1.00
	IV	52	1	0.98
	V	79	5	0.94
	VI	64	26	0.71
	VII	46	14	0.77
	VIII	34	10	0.77
Cemetery	I	4	0	1.00
	II	19	1	0.95
	III	18	0	1.00
	IV	53	0	1.00
	V	79	5	0.94
	VI	64	26	0.71
	VII	45	15	0.75
	VIII	24	20	0.55
Knox County	I	4	0	1.00
	II	19	1	0.95
	III	18	0	1.00
	IV	53	0	1.00
	V	77	7	0.92
	VI	52	38	0.58
	VII	38	22	0.63
	VIII	22	22	0.50
US	I	4	0	1.00
	II	19	1	0.95
	III	18	0	1.00
	IV	53	0	1.00
	V	77	7	0.92
	VI	48	42	0.53
	VII	37	23	0.62
	VIII	22	22	0.50

**Table 4.** Cumulative bionomial tests of the performance of Lovejoy et al. [10] method across the four priors.

*Method*	*Coverage*	*Number of Successes*	*Number of Failures*	*p-value*	*Probability of Success*
Donated Collection	90%	316	57	0.0013	0.8472
Cemetery	90%	306	67	< 0.0001	0.8204
Knox County	90%	283	90	< 0.0001	0.8097
US	90%	278	95	< 0.0001	0.7453

## 4. Discussion

The age-at-death distribution of a skeletal collection, cemetery, county, and country survivorships plotted somewhat similarly as regards to overall shape of the curves, but age differed among the skeletal and cemetery samples vs. the county and country samples. Moreover, there were key shape differences, in particular, at older ages. Despite these differences, the prior samples provided age ranges that were roughly similar, but the skeletal collection (forensic method) generated the best age estimates. Thus, the hypothesis that small differences will be reported that will not greatly impact performance was supported. However, all four prior samples' coverage underperformed, indicating biological age of the sacroiliac joint does not translate linearly to chronological age.

It is not surprising there were large age ranges in advanced phases and the performance of the age ranges was lower than probability; many scholars report unease with using the method [15]–[20]. Despite this, the technique has also enjoyed great success in the literature on Portuguese and Greek populations [2],[17],[19] and overall; the method performed well here with an 85% probability of success. It could be stated that the American population did not perform as well because it may require an informative prior not derived from just a local or national population. Instead, it likely simply requires a population that shares a similar aging progression.

Even though a different population may be needed to more accurately model auricular surfaces in American males, the level of representation of a sample appears to be important. Upon examination of Tables 2–4, performance of the method deteriorates as the informative prior moves from a more local (skeletal collection and cemetery) to a more general level (county and country). This may reflect genetic (genetic drift) or epigenetic differences in aging in the local populations. Genetic and epigenetic effects may have been mitigated in the county data by the larger sample size and greater variation in the county data. As it relates to the local level, while everyone had equal probability of being buried in the local cemetery, plots and mausoleums are often purchased as family units, and thus there may have been a kinship bias introduced from familial mortuary practices.

The trends in the fit of the informative priors support Godde and Hens [1] who detailed there is flexibility in informative prior selection. Additionally, the forensic method (using a sample of the target population as the informative prior) performed slightly better than the other three samples, a finding consistent with Godde and Hens' [4] comparison of the two approaches. Thus, informative priors may best be derived from a portion of the target sample, although there is some flexibility if this is not desirable.

The broad age ranges associated with the sacroiliac aging method indicate that as we transition through the auricular surface phases, aging becomes more variable. Thus, there is a lot of diversity in age-related changes at the sacroiliac joint. This may be due to the normal degeneration of tissue, which is a less reliable process than growth and development progression. Biomechanical forces may also be driving this, although it is less clear how as the sacroiliac joint functions as a stabilizer and load dissipater for the lower extremities from the weight of the trunk [21], a biomechanical function of bipedalism.

These results directly impact practitioners working with aged populations. It is important to understand the increasing variability in presentation of biological age, which translates to greater diversity in how older adults respond to and present age-related changes in the sacroiliac joint. This may also explain some of the difficulty in making diagnoses of specific sacroiliac joint conditions during life [21]. Further, demographic information from medical and anthropological research conducted on a national level may not be highly comparable to a local population of older adults. Practitioners should be cognizant of the differences in level of population analysis when making diagnoses.

These findings can be applied to reconstructing the biological impact of lifeways of historic Americans and identifying individuals in forensic contexts. Only through knowledge of modern Americans with documentation of demographics in life can we learn how to interpret the osteobiography of historic Americans. Modeling age in historic Americans should follow the recommendation of this paper to find a more localized population (i.e., city or a smaller subset of the city level) or to utilize the forensic approach. Moreover, time is an important component; Godde and Hens [4] found a temporal trend in pairing populations for statistical modeling of age indicator data. Forensic anthropologists can use the conclusions of this paper, in combination with the results from other work, to customize the results of their age analysis by applying HPDRs generated using appropriate informative priors.
